# Activity and Toxicity of *Eleutherine palmifolia* (L.) Merr. Extract on BALB/c Mice Colitis-Associated Colon Cancer Model

**DOI:** 10.31557/APJCP.2020.21.12.3579

**Published:** 2020-12

**Authors:** Roihatul Mutiah, Riza Ambar Sari, Wahyi Yucha Firsyaradha, Anik Listiyana, Yen Yen Ari Indrawijaya, Abdul Wafi, Arief Suryadinata, Retno Susilowati, Ana Rahmawati

**Affiliations:** 1 *Department of Pharmacy, Faculty of Medical and Health Sciences, Maulana Malik Ibrahim State Islamic University, Malang, Indonesia. *; 2 *Department of Medical Education, Faculty of Medical and Health Sciences, Maulana Malik Ibrahim State Islamic University, Malang, Indonesia. *; 3 *Department of Biology, Faculty of Medical and Health Sciences, Maulana Malik Ibrahim State Islamic University, Malang, Indonesia. *

**Keywords:** Eleuthrine palmifolia (L.) Merr., colon cancer, TNF-α, TGF-β, hepatotoxicity

## Abstract

**Objective:**

*Eleutherine palmifolia* (L.) Merr. extract (EPE) containing isoliquiritigenin and oxyresveratrol is believed to be an anticancer agent. This study evaluates colon histopathology, TNF-α, TGF-β, and hepatotoxicity on BALB/c mice colitis-associated colon cancer (CAC) model treated with EPE.

**Methods:**

In vivo study was performed on BALB/c mice CAC model induced by 10 mg/kgBW AOM on the first day followed by administration that each cycle consisted of 5% DSS in water for seven days and regular water for seven days. The indicators of the formation of CAC were observed by a fecal occult blood test (FOBT) and serum amyloid α (SAA) test. The treatment was conducted once a week started from the seventh week up to the twentieth week with six treatment groups: I was administrated by regular water only (negative control), II was administrated by AOM and DSS only (positive control), III was administrated by doxorubicin, IV-VI were treated by EPE (0.25 mg/kg BW, 0.50 mg/kg BW, and 1.00 mg/kg BW) respectively. The colon and liver’s histopathology was observed using hematoxylin-eosin (HE) staining, TNF-α with immunohistochemistry (IHC), and level measurement of TGF-β colon with ELISA reader. The data were used one-way ANOVA followed by post hoc as statistical analysis.

**Results:**

The administration of EPE increased the expression of TNF-α, the total of goblet cells of the colon, and decreased the level of TGF-β. Administration of EPE 0.50 mg/20g BW decreased a liver histopathological score but induced a histopathological alteration of the liver at a dose of 1.00 mg/20g BW.

**Conclusion:**

This study indicate that EPE could be recommended as a colon anticancer through increase the goblet cells, induce apoptosis through increase TNF-α, and decrease TGF-β.

## Introduction

Colorectal cancer is the second fatal cause of death for men and third cause of death for women. According to the Global Burden of Cancer (GLOBOCAN) 2013, there are around 12.8 people among 100.000 people in Indonesia that may suffer from colorectal cancer with mortality rate 9.5% of all cancer disease. 

The risk factors of colon cancer were increased with colitis. Ulcerative colitis (UC), a chronic disease in colon mucosa is commonly caused by the interaction of several factors including environmental, genetic, and immunologic (Rogler, 2014). The mutational inactivation of TGF-β that combines with tumor suppressor adenomatous polyposis coli (Apc) in the epithelial intestinal cell is develops invasive adenocarcinomas (Calon et al., 2012). Finally, the active TGF-β is fewer, thus it cannot do its physiological function to pressing T-cell proliferation (Guda et al., 2003). 

Moreover, the MUC2 expression from goblet cells is obstructed by Wnt/β-catenin in colon cancer. As a result, about 80-90% of colon cancer is histologically characterized by tissue without any mucin. While, goblet cells excreting the mucin compound, particularly MUC2 acts as a barrier to the mucosa (Van Raay and Allen-Vercoe, 2017). 

*P53* gene mutation also happens in colon cancer, so it will not be able to do apoptosis induction. One of the indicators to know the occurrence of apoptosis is TNF-α. In the body’s immune system, TNF-α plays a crucial role as an antitumor since it can trigger apoptosis and antiproliferation to fight cancer cells (Tyasmara and Sandhika, 2018).

It is well known that Indonesia rich of biodiversity. Around 30,000 types of plants exist and 7,000 of them are promising as medicinal plants. The safety of herbal medicine needs to be confirmed before the treatment. The vital phase in ensuring safety is a toxicity test. One of the organs that working as a poison or toxic substance detoxifier is the liver. The accumulation of toxic substances in liver parenchyma may damage hepatocyte and cause various histopathological changes. One of the plants having the potential as medicine is *Eleutherine palmifolia* (L.) Merr.). Empirically, *Eleutherine palmifolia* (L.) Merr.) is effective to cure skin disease, cough, and bloody diarrhea. In addition, it also can be used as a laxative, diuretic, and analgesic. The former study proves that *Eleutherine palmifolia* (L.) Merr also acts as a breast anticancer, cervical anticancer, and colon anticancer (Li et al., 2009).

These findings are very important, as it has been speculated that EPE may contribute to the colon anticancer. This study employs BALB/c mice of colitis-associated colon cancer model induced by azoxymethane (AOM) and dextran sodium sulfate (DSS). AOM has been found to be more stable in solution than 1,2-dimethylhydrazine (DMH). The combination of AOM and DSS has gained popularity for its reproducibility, potency, and develop adequate tumors in as 7-10 weeks than other models that require several months (Thaker et al., 2012). This study has analyzed the effect of *Eleutherine palmifolia* (L) Merr extract on colon histopathology, TNF-α expression, TGF-β level, and its hepatotoxicity on BALB/c mice of colitis-associated colon cancer model.

## Materials and Methods

*Plant material*

*Eleutherine palmifolia* (L) Merr. bulbs were obtained from Central Kalimantan province, Indonesia. The plant was identified and authenticated by Materia Medika Batu, East Java, Indonesia (No.074/348/102.7/2017), and then powdered. The identified specimens were stored in Pharmacognosy Laboratory, Maulana Malik Ibrahim State Islamic University of Malang.

*Experimental Animals*

36 female BALB/c mice were purchased from the Faculty of Veterinary Medicine, Airlangga University at 8-10 weeks of age and 20-30 grams of weight. The animals were acclimatized for a week prior to the experiment. The controlled environment was maintained into a dark-light cycle (12:12 h). The mice were given standard pellets, and drinking water ad libitum This animal study was approved by Polytechnic of Health, Ministry of Health, Malang with ethical approval number 027/KEPK-POLKESMA/2019. 

*Plants extraction*

*Eleutherine palmifolia* (L.) Merr. bulbs were dried at a temperature of 40°C in an oven up to the moisture content less than 10%. The bulbs were macerated with 96% ethanol pro analysis (Merck KgaA, Darmstadt, Germany) using ultrasonic-assisted extraction (UAE) then filtered. Afterward, the filtrate was evaporated at 40°C using a vacuum rotary evaporator and kept in the oven until a thick extract was obtained. 

*Colitis-associated colon cancer model*

Female BALB/c mice were injected intraperitoneally with 10 mg/kg BW of colonic carcinogen, AOM (Sigma Aldrich, UK) on the first day. Afterward, the mice were administered with 5% (w/v) DSS (Mr=15.000, Sigma Aldrich UK) in water for seven days, followed by another week of regular water. In the sixth week, a fecal occult blood test (FOBT) and serum amyloid α (SAA) test were performed. The examination of FOBT using both macroscopically and microscopically to detect the bleeding stool that may indicate colorectal cancer. The macroscopically blood detection in FOBT involves smearing some stool onto absorbent paper. Afterward, benzidine was dropped onto the stool specimens. The blood was presented if the paper changed into green color. While, the microscopically blood detection by examined the smearing feces onto objective glass then observed the erythrocyte with the light microscope (Simadibrata, 2010). The SAA test was analyzed with a serum which collected from mice of each group then examined with mouse SAA immunoassay ELISA kit (Dupaul-Chicoine et al., 2010)

**Figure 1:**
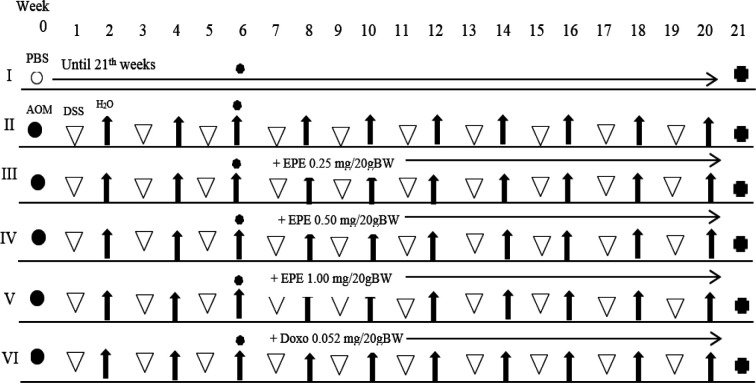
The Experiment Design of Mice Induced with AOM and DSS as Colitis-Associated Colon Cancer (CAC) Model and EPE Administration. Notes: **O**= PBS, • = AOM, = DSS, ↑ = water (H_2_O), = SAA & FOBT test, = sacrificed

In the seventh up to the twentieth week, 0.5 ml EPE therapy was administrated by oral based on therapy groups (Endharti et al., 2016). 0.5 ml doxorubicin administration was conducted in the seventh up to twentieth week by injected intraperitoneally. Based on previous study EPE doses are 0.25 mg/20g BW, 0.50 mg/20 g BW, and 1.00 mg/20g BW. The CAC model experimental design is presented in Figure 1. 

*Colon histopathological analysis*

The colon tissue was cut and washed in ice-cold PBS then visualized by hematoxylin-eosin (HE) staining using the paraffin method. Colon specimens were observed using light microscopy (Olympus) in 10 visual fields (400 x magnification) randomly count the total goblet cells in the colon mucosa (Wicaksono and Permana, 2013).

*TNF-α expression analysis*

Immunohistochemistry staining was performed using paraffin block fixation of neutral buffered formalin. The primary antibody employed was TNF-α polyclonal antibody (rabbit polyclonal anti-mouse TNF-α) (Dako Glostrup, Denmark), biotin-conjugated anti-rabbit secondary antibody (Dako Glostrup, Denmark). The reaction of anti-TNF-α antibody and diaminobenzidine (DAB) chromogen (Sigma Aldrich, UK) visualized brown patterns. Then, the expressions were evaluated by light microscopy (Olympus) in 10 visual fields (400 x magnifications).

*TGF-β level analysis*

The TGF-β level was measured using the manufacturer’s instruction of a mouse TGF-β ELISA kit. The evaluation was conducted in Biology Genetic Laboratory, Maulana Malik Ibrahim State Islamic University Malang. The colonics protein was extracted by lysing cells through a homogenous process with RIPA buffer (Cell Signaling Technology Cat no.9806) diluted with ddH2O which was added with protease inhibitor and phenylmethylsulfonyl fluoride (PMSF). 

*Liver histopathological analysis*

Histological analysis of liver measured based on the score of liver cell histopathological changes by modification of Manja Roenigk. Those are normal (score 1), parenchymatous degeneration (score 2), hydropic degeneration (score 3), and necrosis (score 4) (Insani and Berata, 2015).

*Statistical analysis*

The experimental results were reported as mean ± standard deviation (SD) of four replicates. The statistical analysis was evaluated by a one-way analysis of variance (ANOVA) followed by post hoc using SPSS 23.0 (Endharti et al., 2016).

## Results

*AOM and DSS Induction as a colitis-associated colon cancer model*

We used AOM followed by repeated DSS administration to developed adequate colitis-associated colon cancer within 20 weeks. They form colon cancer in the middle to the distal of mice. AOM cause deoxyribonucleic acid (DNA) cells damage because of *Apc* gene mutation, K-Ras pathway, p53, and β-catenin increase. Repetitive administration of DSS causes inflammation on colon epithelium leading to an increase of colitis (Fazio et al., 2011). In this work, the EPE therapy was performed after three cycles of DSS administrations. The EPE doses were 0.25 mg/20g BW, 0.50 mg/20g BW, 1.00 mg/20g BW for group IV, V, and VI, respectively (Endharti et al., 2016). The results of the serum amyloid α (SAA) test and fecal occult blood test (FOBT) in the sixth week showed a significant difference between positive control (AOM/DSS only) and negative control (normal) shown in Table 1. 

*The total goblet cells of the colon in BALB/c mice of CAC model*

**Figure 2:**
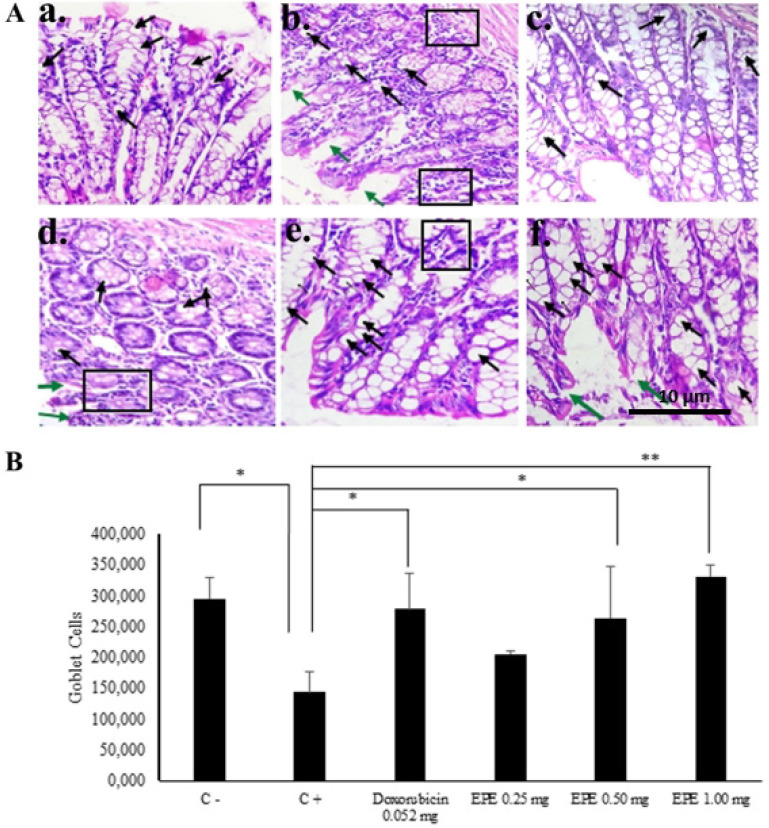
A. Goblet cells histopathology of the colon of BALB/c mice of CAC model after EPE treatment. HE staining. (a) Positive control, (b) Negative Control, (c) Doxorubicin of 0.052 mg/20gBW, (d) EPE of 0.25 mg/20gBW, (e) EPE of 0.50 mg/20gBW, (f) EPE of 1.00 mg/20gBW. 400x magnification. Notes: →, goblet cells; → , epithelium damage, , inflammatory cells infiltration. B. Goblet cells total graph. *p<0.05; **p<0.01

Adequate goblet cells thus produce mucin (MUC2) in the colon are critical to the prevention of colitis. Colon histology showed that many more goblet cells were retained in EPE-treated mice compared with those treated AOM/DSS only (Figure 2A). In mice treated AOM/DSS only, we found epithelium damages and inflammatory cell infiltrations was occur. In addition, the morphological differences of inflammatory and goblet cells were found in the various of EPE doses. It was indicated on the total goblet cells in the colon mucosa. The total of goblet cells increased upon the rising of EPE doses where the EPE 1.00 mg/20 g BW exhibited the highest total goblet cells than the positive control (p=0.000) (Figure 2B). 

*TNF-α expression in BALB/c mice of the CAC model*

**Figure 3:**
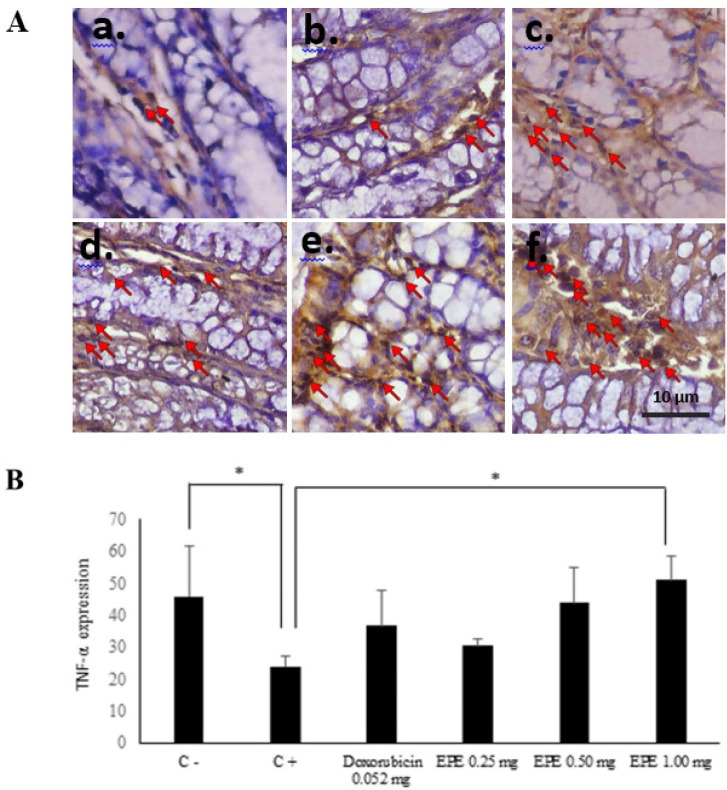
A. The TNF-α IHC staining of colon tissue CAC model with 400x magnification. (a) Positive control, (b) Negative Control, (c) Doxorubicin of 0.052 mg/20gBW, (d) EPE of 0.25 mg/20gBW, (e) EPE of 0.50 mg/20gBW, (f) EPE of 1.00 mg/20gBW. Notes: → TNF-α expression (apoptosis). B. TNF-α expression graph. * p<0.05

The administration of AOM and DSS significantly could decrease TNF-α expression in the colon (p= 0.045). Compared to group II (AOM/DSS induction), EPE administration for group IV (EPE 0.25 mg/20g BW) p=0.923, group V (EPE 0.50 mg/20g BW) p=0,084 and group VI (EPE 1.00 mg/20g BW) p=0.010 can increase TNF-α expression. A significant increase of TNF-α expression was exhibited in the EPE dose of 1.00 mg/20g BW. The results of TNF-α expressions were presented in Figure 3.

*TGF-β level in BALB/c mice of the CAC model*

**Figure 4:**
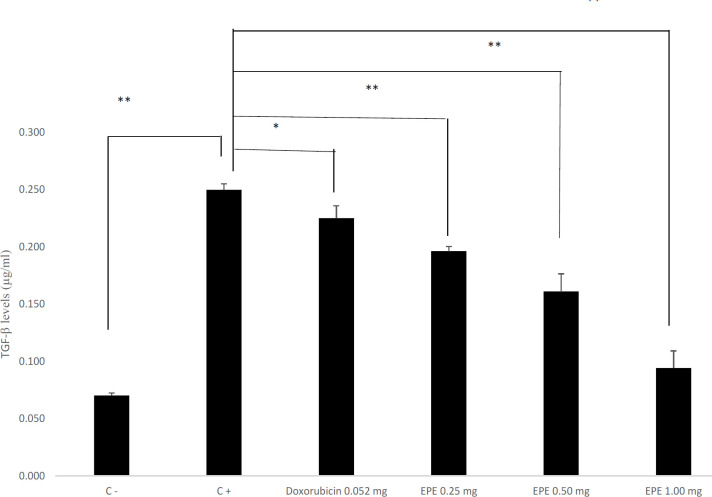
EPE Decrease TGF-β Levels in BALB/c Mice Colitis-Associated Colon Cancer (CAC) Model. The result shown mean+SD, with 4 replicates in each group. *p<0.05,**p<0.001

TGF-β level in AOM and DSS induction groups experienced a significant increase (p=0.000). EPE administration in all doses dramatically decreased the TGF-β level with the p-value of 0.031, 0.000, 0.038 for group III (0.25 mg/20g BW), IV (0.50 mg/20gBW), and V (1.00 mg/20g BW), successively. The TGF-β level decreased mostly in a dose of 1.00 mg/20g BW. The measurement of TGF-β level by using the ELISA technique is shown in Figure 4. 

*The liver histopathological score of the CAC model*

The observed liver histopathology parameters were normal hepatocyte, parenchymatous degeneration, hydropic degeneration, and necrosis. The scoring was summarized after obtaining percentages of every parameter as shown in Table 2. 

**Figure 5:**
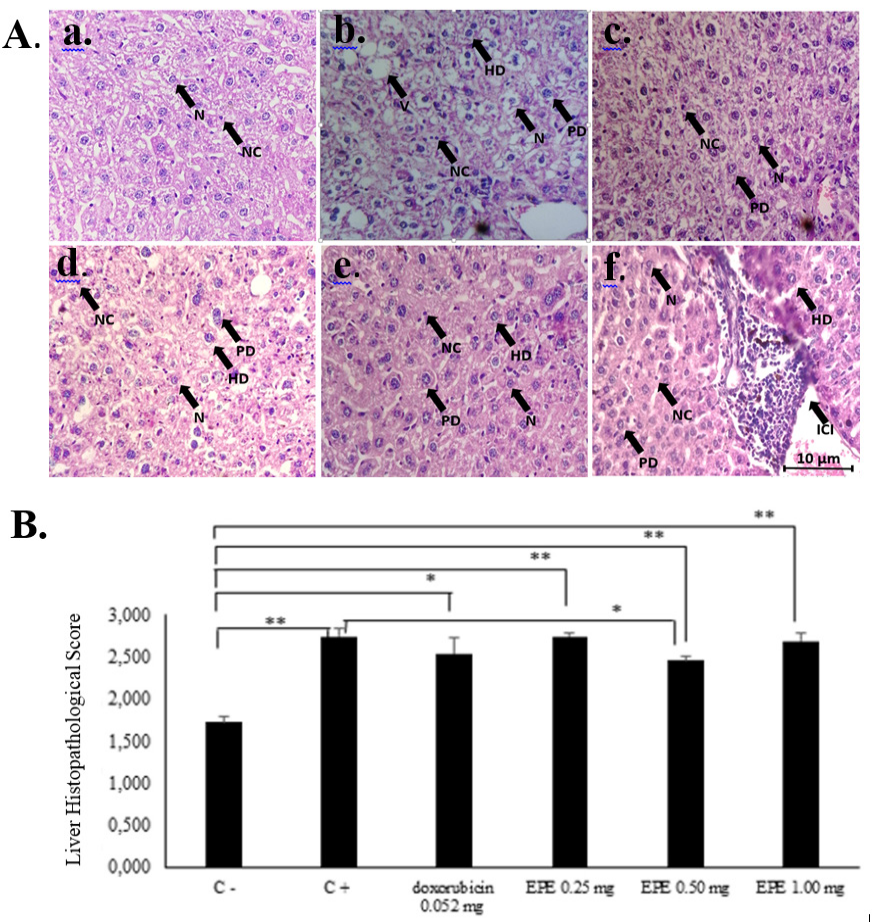
A. The illustration of liver histopathological in BALB/c mice of colitis-associated colon cancer (CAC) model. HE staining. 400x magnification. Notes: N, normal hepatocyte; PD, parenchymatous degeneration; HD, hydropic degeneration; NC, necrosis; V, vacuolization; ICI, inflammatory cells infiltrations; B, Liver histopathological graph of BALB/c mice of CAC model. * p<0,05, ** p<0,001

EPE dose of 0.25 mg/20gBW was assumed incapable of giving hepatoprotective effect as seen from histological analysis compared with the positive control (AOM/DSS induction) (Figure 5). EPE of 1.00 mg/20g BW, a high dose, could harm and cell death. Thus, it could damage liver histopathology characterized by the inflammation (Hwang et al., 2018; Kumar et al., 2013). 

## Discussion

BALB/c mice induced by AOM carcinogenesis might penetrate into cells and metabolism processes by enzyme phase I that produced reactive metabolite. This metabolite is a radical compound that easily binds to DNA, so it causes gene mutation. DNA damage and the gene mutation is the first phase of initiation in cancer cell development. The induction of AOM reported have a detoxification process in the liver performed by microsomal enzymes. Methylazoxymehanol (MAM) compound was formed in the first phase by cytochrome P-450 (CYP-4502EI) enzyme through several phases of N-oxidation and hydroxylation (Rosenberg et al., 2008). While, the CAC model induced by 5% DSS shows inflammation that affects epithelial damage and decreases mucin secretion (Hwang et al., 2017). Anthracyclines have been shown to inhibit tumor cell growth in several tumor lines and neoplasm. The previous study reported that a single injection of doxorubicin increases apoptosis in the stem cell zone of the jejunum, followed by mucosal damage involving a decrease in crypt proliferation, crypt number, and villus height. The intestinal mucosa repair occurred and return to normal morphology characterized by crypt hypertrophy and paneth cell hyperplasia (Rachael et al., 2016). Besides, using a low dose of doxorubicin could minimize the side effect. ASCO has developed guidelines for the prevention and monitoring of cardiac dysfunction of adult survivors of cancer. According to the guidelines, the risk of cardiac dysfunction is increased with the following high dose anthracycline therapy (eg, doxorubicin >250 mg/m^2^) (Armenian, 2017). 

In our previous study, there were two compounds, Oxyresveratrol and, Isoliquiritigenin that identified by 96% ethanol pro analysis of *Eleutherine palmifolia* (L.) Merr extract. Both compounds were believed to lead compounds in anticancer activity (Mutiah, 2019). Isoliquiritigenin is one of the compounds categorized as a flavonoid group. This compound has antioxidant, anti-inflammatory, and antiplatelet activities. A study related to an inflammation on HT-29 cell reported that isoliquiritigenin can suppress inflammatory mediator expressions such as cyclooxygenase-2 (COX-2) and intercellular adhesion molecule (ICAM-1) (Jin et al., 2016). The other compound is oxyresveratrol that has strong anti-inflammatory and antioxidant activities. During the inflammation process, pro-inflammation mediators such as nitric oxide (NO) and PGE2 increased. Oxyresveratrol can significantly decrease the pro-inflammation level of NO, PGE2, IL-6, and MCP-1 on mice induced by DSS 2% (Hwang et al., 2017). Oxyresveratrol has a certain function for reducing cell proliferation up to 5%. Resveratrol interacted with cytochrome p450 isoenzymes hence it can inhibit and decrease COX2 that caused inflammation. This compound is also able to reduce the NF-κB transcription factor bind to DNA which is usually increased in cancer development. A previous study pointed out that resveratrol metabolites can inhibit metastases of cancer cells as well as able to induce cell death (Aires et al., 2013). 

EPE therapy was effective in the increasing total of goblet cells. It can be associated with the high compound contained in EPE. One of them is oxyresveratrol (Mutiah, 2019), a compound that is responsible to produce mucin (particularly MUC2) and leading to increase in the total goblet cells. MUC2 function is as a barrier to protect the epithelium from bacteria (Hwang et al., 2017). The mechanism of oxyresveratrol compound found in EPE can also be associated with the increasing NAD+ synthesis. In a normal condition, NAD+ becomes a coenzyme in a redox reaction and decreasing total of NAD+ is effective for increasing oxidative stress. Oxidative stress increasing in the colon can initiate the formation of colitis. Oxyresveratrol contained in EPE plays a role as a strong antioxidant and the anti-inflammatory activity decreases the severity of colitis. Oxyresveratrol increases NAD+ by stimulating enzyme that taking part in NAD+ biosynthesis. NAD+ synthesis stimulated by oxyresveratrol performed in all pathways. Then, decreasing oxidative stress causes MUC2 secretion tp increase the total of goblet cells (Hwang et al., 2018).

The total of goblet cells in mice treated AOM/DSS only was a bit, it might be bacteria expansion has been associated with the total goblet cells thus secreted MUC2 protein was lower. The mucin has a highly O-glycosylated protein, forming large net-like structures. The integrity and permeability of colon were interrupted by microbial translocation or entering bacteria to the mucosal barrier. It is constructed by epithelial cells to maintain homeostasis by segregating microbiota and immune cells. Impaired mucosal barrier contributes to the development of inflammation (Okumura and Takeda, 2018). Disruption of epithelium characterized by mucosal inflammation in lamina propria. The activation of neutrophils that produce IL-1β which leads to dendritic and macrophage produce IL-6, a cytokine that acts as a tumor promoter (Wang et al., 2014). Macrophage was an important proinflammatory cytokine that regulates epithelial barrier function while neutrophil was promoted proliferation and tissue damage (Kiesler et al., 2015). 

In TNF-α expression, the histological analysis of mice induced by AOM/DSS caused disturbance of their cell regulation then the proliferation will increase and cell apoptosis will decrease. The cancer chronic condition caused a pressuring effect on TNF-α, leading to a lack of TNF-α and TNF-α inhibition. Thus, the expression of TNF-α was low (Tyasmara and Sandhika, 2018).

A result of the study showed that EPE could increase apoptosis activity through cancer cells which is characterized by increasing TNF-α expression. TNF-α interacts with two types of cell surface receptors types I and II to regulate various cell type specific responses. Activated TNFRI recruits TRADD (TNFR-associated death domain), which in turn recruits TRAFs (TNFR-associated factors) and RIP to activated NF-κB. TNFRI also stimulates the formation of cytoplasmic TRADD complex, containing FADD (FAS-associated death domain) and pro-caspase-8, leading to the activation of caspase-8 and the initiation of an apoptotic signaling cascade. Isoliquiritigenin could induce TNF-α through autophagy on cell S180 (Yushan et al., 2018). 

TGF-β is an immunosuppressive cytokine that contains an anti-proliferative effect in tumor cell development as well as induces apoptosis in normal cells. The anti-proliferative effect is intermediated by TGF-β type I and II receptors forming dimer and eventually, it activates the transcriptional regulatory protein of SMAD, SMAD2, 3, and 4. Immunosuppressive function in the tumor was frequently disrupted during the cancer cells transformation process as the effect of mutation or disappearance of gene expression from one component or more of TGF-β pathways (Calon et al., 2012; Rosenberg et al., 2008). In colon cancer, carcinogenesis occurred through silencing several genes involved in DNA mismatch repair that causes a frame-shift mutation in TGF-βRII. A previous study declared that mice induced by AOM have TGF-β1 signal dysfunction, thus, they have a high level of ligand (Guda et al., 2003).

A high percentage of the whole ligands of the TGF-β1 after AOM administration is not comparable with the active form of TGF-β1 where the level is 30% lower. It is caused by mutational inactivation of TGF-β signaling pathway during colon cancer development (Guda et al., 2003). Functionally, TGF-β inhibited proliferation and tumorigenesis process; however, during colon cancer cells development, TGF-β signaling was to suppress the effect of tumor suppressor so that TGF-β (TGFβR1 and TGFβR2) or intracellular mediator of SMAD (SMAD4, SMAD2, and SMAD3) were failed in inactivating cancer cells (Yushan et al., 2018).

EPE of 1.00 mg/20gBW contained high phenolic hydroxyl group (-OH) which has oxidative stress properties that give cytotoxic activity may lead the liver histopathological damage when given for long periods and high dose (Wulandari et al., 2017). The phenolic hydroxyl group (-OH) plays an important role in uncoupling of the respiratory chain in the mitochondria. Presence of injury in mitochondria causes decrease in ATP production so that the damaged cell condition depends on oxidative metabolism in mitochondria (Kumar et al., 2013). EPE of 0.50 mg/20gBW showed a good response hence it could repair liver histopathology caused by AOM induction. It was characterized by a decreased in the histopathological score. It can be assigned to antioxidants such as oxyresveratrol contained in *Eleutherine palmifolia* (L.) Merr. It was able to decrease the histopathological score. This dose was more effective than EPE of 0.25 mg/20gBW and 1.00 mg/20gBW. The effective dose of 0.5 mg/20gBW in line with the result of the previous study related to IC_50_ of *Eleutherine palmifolia* (L.) Merr. ethanol extract on WiDR colon cancer cells that have IC_50_ 104.52 μg/ml (Mutiah, 2019). The result of IC_50_ has been converted into the dose for mice that is 0.50 mg/20gBW. The illustration of liver histopathology is shown in Figure 5 A. 

Oxyresveratrol in *Eleutherine palmifolia* (L.) Merr. was effective in giving protective effects for the liver against oxidative, inflammatory and antiapoptosis stress in hepatocyte. Oxyresveratrol can play a role as hepatoprotective and anti-inflammatory by blocking phosphorylation in the NF-κB pathway and Keap1-Nrf2 pathway activation (Ahmad et al., 2018).

In conclusion, EPE attenuates inflammatory by increasing the total goblet cells of the colon, induces apoptosis through increased expression of TNF-α and decreased TGF-β level. Histological analysis of mice induced AOM/DSS indicates that EPE might reduce the severity of colitis. However, these findings suggest that the best dose based on liver histopathological analysis that might have functioned as a hepatoprotection is EPE 0.50 mg/20g BW.

**Table 1:** Result of SAA and FOBT for Colitis Test

Group	SAA Concentration (ng/ml) ± SD	Total erythrocytes of FOBT	
		Micro	Macro
Control (-)	107.30 ± 2.60	2 ± 0.81	-
Control (+)	359.35 ± 3.29*	39 ± 2.16*	+
Doxorubicin 0,052 mg/20gBW	348.59 ± 1.67 *	37 ± 1.63*	+
EPE 0.25 mg/20gBW	346.63 ± 3.78*	32 ± 1.71*	+
EPE 0.50 mg/20gBW	330.75 ± 4.22*	35 ± 2.08*	+
EPE 1.00 mg/20gBW	357.62 ± 9.16*	42 ± 1.71*	+

*p < 0.01 higher significant different than the control (-) group

**Table 2:** The Percentage of Liver Histhopathological Damage of BALB/c Mice Colitis-Associated Colon Cancer (CAC) Model

Group	Parameter				Total Score ± SD
	Normal	Parenchymatous Degeneration	Hydropic Degeneration	Necrotic	
C (-)	70.06%	8.27%	0.58%	21.09%	1.727 ± 0.072
C (+)	31.63%	14.71%	1.16%	52.50%	2.745 ± 0.089
Doxorubicin 0.052 mg/20gBW	38.05%	14.99%	1.56%	45.39%	2.543 ± 0.193
EPE 0.25 mg/20gBW	30.96%	15.57%	1.60%	51.87%	2.744 ± 0.037
EPE 0.50 mg/20gBW	42.41%	12.81%	0.79%	43.98%	2.464 ± 0.052
EPE 1.00 mg/20gBW	36.83%	9.69%	1.20%	52.28%	2.689 ± 0.093
